# Live imaging of the *Drosophila* ovarian germline stem cell niche

**DOI:** 10.1016/j.xpro.2021.100371

**Published:** 2021-03-05

**Authors:** Scott G. Wilcockson, Hilary L. Ashe

**Affiliations:** 1 Faculty of Biology, Medicine and Health, University of Manchester, Manchester M13 9PT, UK

**Keywords:** Cell Biology, Microscopy, Model Organisms, Stem Cells

## Abstract

The maintenance of stem cell populations and the differentiation of their progeny is coordinated by specific communication with associated niche cells. Here, we describe a protocol for short-term live imaging of the *Drosophila* ovarian germline stem cell niche *ex vivo.* By immobilizing the ovarian tissue in a fibrinogen-thrombin clot, we are able to maintain the tissue for short-term high-temporal live imaging. This enables the visualization of dynamic cellular processes, such as the cytoskeletal dynamics that control stem cell niche communication.

For complete details on the use and execution of this protocol, please refer to Wilcockson and Ashe (2019).

## Highlights

• *Drosophila* ovarian germaria can be imaged live with high-temporal resolution *ex vivo*
• The dissected ovarian tissue is immobilized in a fibrinogen-thrombin clot
• Germline stem cell-niche interactions are visualized by live confocal microscopy
• This approach enables the study of ovarian germline and somatic cell dynamics

## Graphical abstract

**Figure:**
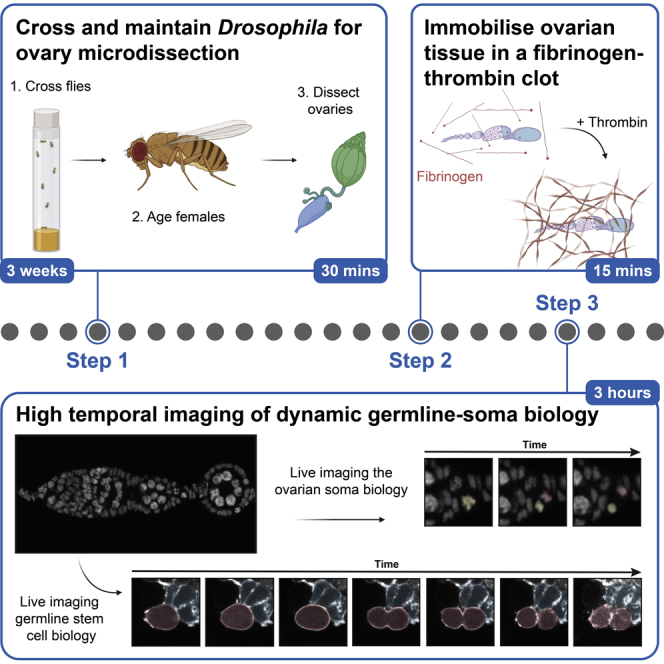

## Before you begin

### Experimental design consideration

The *Drosophila* ovarian germline provides a well-studied model of stem cell biology and the mechanisms that regulate their self-renewal and differentiation; from stem cell niche communication and post-transcriptional regulation to systemic signaling and aging. While the majority of this work to date has relied on “snap-shot” views of GSC biology, live imaging of the ovary is now revealing the cell biology that contributes to GSC self-renewal (Wilcockson and Ashe, 2019) and daughter cell differentiation (Chanet and Huynh, 2020).

*Drosophila* has represented a premier model for investigating developmental cell biology *in vivo* for decades and this has resulted in the generation of a multitude of fluorescent reporters, particularly based on the Gal4-UAS system (Brand and Perrimon, 1993). Many of these constructs rely on a UASt promoter which drives transgene expression in somatic tissues (including that of the ovary), however, this promoter drives very poor expression in the germline. This was subsequently altered by the addition of a transposase promoter element expressed in the female germline to create UASp (Rorth, 1998). This created a promoter that drives high expression in the germline but not in somatic tissues. Most recently, DeLuca and Spradling (2018) resolved this final issue with the creation of UASz which drives higher expression than UASp in the germline and equally high expression as UASt in somatic tissue. Unfortunately, this has yet to be adopted by the community as a default promoter, therefore promoter choice will need to be considered when choosing established reporters and when creating new lines.

A second consideration, that is important for any experiment, is the choice of reporter used. Different methods of labeling cellular compartments and structures can have deleterious effects. For example, different actin labels expressed in developing egg chambers were found to cause aberrant actin dynamics (Spracklen, et al., 2014). While this is partly explained by the affinity of different reporters to actin, the reporter was also sensitive to temperature and the strength of the Gal4 driver used. It is therefore important to empirically test new reporters, for example, by testing fertility rates in transgenic lines.

A final consideration when studying GSC biology, is to keep in mind their regulation at the systemic level. Reproductive organs are plastic and respond to many signals, including nutrition, and hormonal cues associated with mating and aging (Drummond-Barbosa, 2019; Ishibashi et al. 2020). It is therefore important to finely control aspects of the flies’ lives that could introduce noise, for example, maintaining experimental females with small numbers of wildtype males (see below) to promote reproduction but prohibit overcrowding (Joshi et al., 1998).

### Preparing flies

**Timing: 3 weeks**

The protocol here describes the experimental setup used to image GFP-Tubulin in the germline but can be applied to other transgenic fly lines as discussed in Expected Outcomes. All *Drosophila* lines are kept at 18°C for long-term maintenance and are kept at 25°C when crossing two lines together. Flies are raised on standard fly food (Table 1).

1. Transfer 20–30 female and 5–10 male *nos-Gal4::VP16, UASp-eGFP.αTubulin84B* flies to a new vial with fresh yeast paste.
2. Maintain the flies at 25°C and flip to a new vial every day.
3. Keep the old vials and incubate them at 25°C to collect emerging virgin female flies and young males for experimental crosses.

***Note:*** The *nos-Gal4::VP16; UASp-eGFP.αTubulin84B* line (BDSC_7253) used in Wilcockson and Ashe (2019) stably expresses *eGFP-Tubulin* in the germline and both transgenes are present on the same chromosome.

***Note:*** If combining multiple reporters or lines (e.g., UASp-RNAi) for experimental comparisons then it will be necessary to establish healthy stocks of double or more balanced lines prior to starting.

**Table 1:** Ingredients for the fly food used in this study

Fly food ingredients	Quantity
Yeast	50 g/L
Glucose	78 g/L
Maize flour	72 g/L
Agar	8 g/L
Nipagen Nipagin	27 mL/L
Propionic acid	3 mL/L

### Preparing media

**Timing: ∼30 min**

4. Supplement Schneider’s Insect Media with 10% FBS, 1% penicillin/streptavidin (SIM).
5. From this stock, make additional stocks:
  a. Supplemented with 10 mg/mL fibrinogen (fSIM)
  b. Supplemented with 200 μg/mL human insulin (iSIM)

***Note:*** fSIM can be aliquoted and stored at −20°C. iSIM should be made fresh on the day.

## Key resources table

**Table:** 

REAGENT or RESOURCE	SOURCE	IDENTIFIER
**Chemicals, peptides, and recombinant proteins**		

Fetal bovine serum	Sigma	F3018
Penicillin-streptomycin	Sigma	P0781
Human insulin	Sigma	I2643
Schneider's *Drosophila* medium	Thermo Fisher	21720-024
Fibrinogen, bovine plasma	Millipore	341573
Thrombin protease	GE Healthcare Lifesciences	27-0846-01
Yeast	Merck	YSC2
Glucose	Fisher Scientific	10385940
Maize flour	Merck	BCR377
Agar	Melford Biolaboratories	A20020-500.0
Nipagin	Merck	H5501
Propionic acid	Merck	81910

**Experimental models: organisms/strains**		

*D. melanogaster; y[1] w[67c23]*	Bloomington	RRID:BDSC_6599
*D. melanogaster*; *w[∗]; GAL4::VP16-nos*	Bloomington	RRID:BDSC_4937
*D. melanogaster*; *M{w[+mC]=UASp-LifeAct.mGFP6}ZH-2A, w[∗]*	Bloomington	RRID:BDSC_58717
*D. melanogaster*; *w[1118]; P{w[+mC]=GAL4::VP16-nos.UTR}CG6325[MVD1], P{w[+mC]=UASp-GFPS65C-alphaTub84B}3*	Bloomington	RRID:BDSC_7253
*D. melanogaster; w[∗]; P{w[+mC]=His2Av-mRFP1}II.2*	Bloomington	RRID:BDSC_23651

**Software and algorithms**		

Fiji	(Schindelin, 2012)	RRID: SCR 002285
GraphPad Prism 7	GraphPad Software	RRID: SCR 002798

**Other**		

PYREX 9 depression glass spot plate	Corning	7220-85
Forceps Watchmaker SS	Scientific Laboratory Supplies	INS4366
FluoroDish cell culture dish 35 mm, 10 mm well	World Precision Instruments	FD3510-100
Leica TCS SP8 AOBS inverted microscope	Leica	NA

## Step-by-step method details

### Maintaining F0 females flies prior to dissection

**Timing: 1–7 days**

1. Collect and set up a cross of 20–30 virgin females and 5–10 males at 25°C in a vial with standard fly food.
2. Transfer flies to a new vial every 1–2 days until the day of dissection to ensure flies do not become immobilized on wet yeast.

**CRITICAL:** If including genetic perturbation, RNAi or transgene expression that could possibly also affect male reproduction, ensure that the males kept with the females to be imaged are wildtype. This is to negate any compounding effects on male reproductive health that might indirectly affect female reproductive health.

***Optional:*** The food can also be supplemented with dry yeast as this is believed to boost reproduction.

***Note:*** Here flies are maintained for 3–5 days prior to dissection to ensure enough time for mating and healthy reproduction but not long enough for the negative effects of aging. This can, however, be adjusted to enable investigation of reproduction and/or aging on GSC biology.

***Note:*** When combining live imaging with RNAi or other genetic perturbations, it may be necessary to raise the flies at different temperatures. For example, increasing the temperature to 29°C may enhance RNAi knockdown. This will need to be determined empirically for each experiment and the temperatures described here will need to be altered to reflect this.

### Ovary dissection and mounting

**Timing: 30–60 min**

3. Preheat 5 mL SIM, 1 mL fSIM and 2 mL iSIM to 37°C.

***Note:*** This step is to stop the precipitation of fibrinogen in fSIM following thawing. We also warm all media used to prevent any dramatic temperature changes during washing and mounting of dissected tissue.

4. Prior to ovary dissection, set up a 6-well plate or glass dissection dish with 3× wells with 500 μL SIM for fly washes prior to dissection, 2× wells on a glass dissection dish with 500 μL SIM for dissection and a single well with 200 μL fSIM.
5. Anesthetize the flies by pumping CO_2_ into the vial or incubating at 4°C for 5 min or until they fall to the bottom of the vial and take 2–3 females.

**CRITICAL:** Take care to ensure the flies do not get stuck in yeast paste as they fall when anesthetized.

6. Euthanize the flies by submerging them in 70% ethanol for 1 min.

**CRITICAL:** Take care not to injure the fly abdomen or submerge them in ethanol for too long to avoid alcohol toxicity.

**Figure 1:**
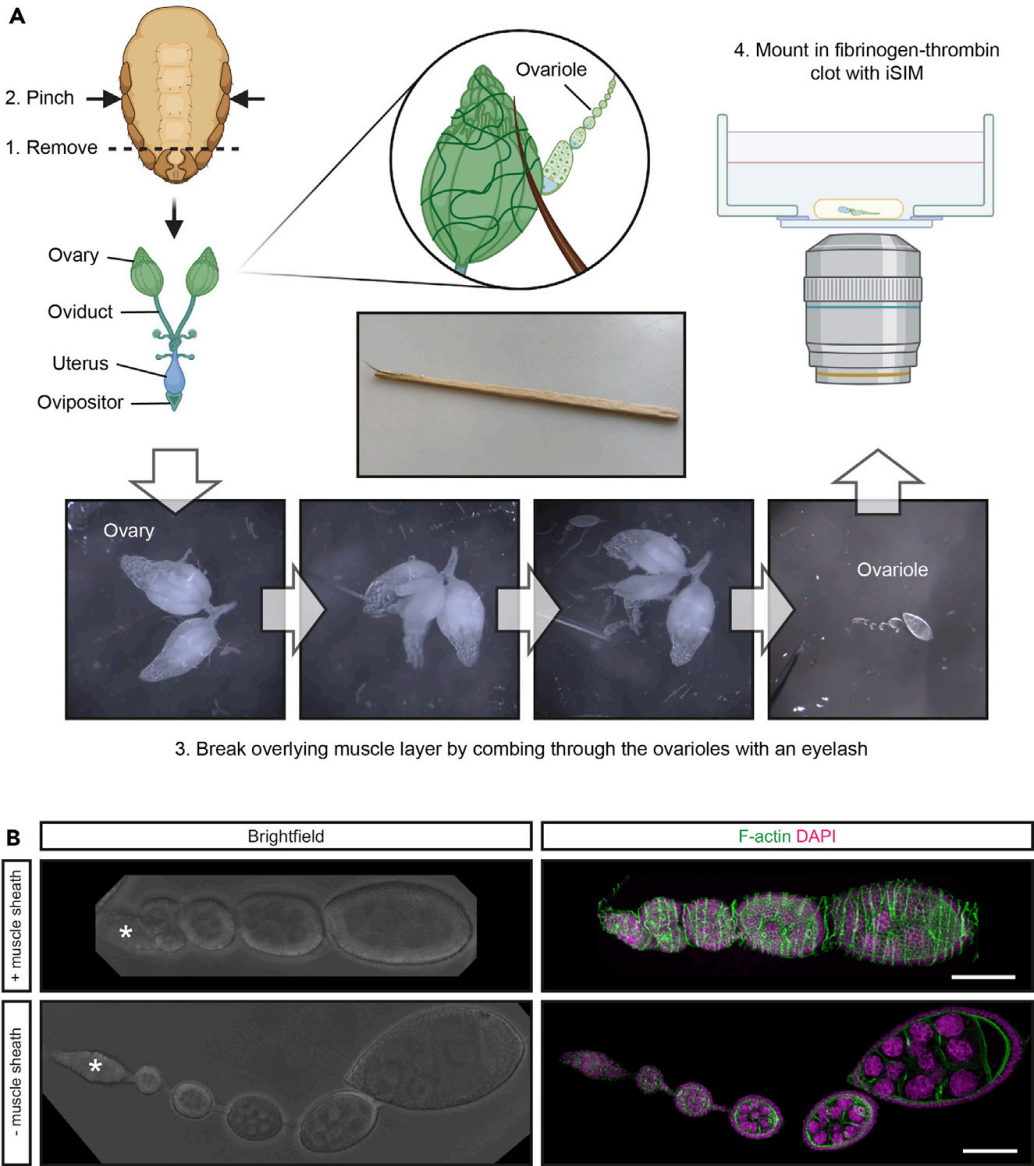
Ovary microdissection (A) Schematic illustrating the dissection of the *Drosophila* ovary and microdissection of the ovarioles for live imaging. (Centre) Image of an eyelash tool. (B) Bright-field and confocal images of ovarioles with and without the overlying muscle sheath. Phalloidin is used to label the F-actin-rich transverse muscle fibers and DAPI labeling of DNA is used to visualize the germline and somatic cells of the ovariole. An asterisk labels the germarium. Scale bar, 50 μm.

7. Transfer the flies to the dissection dish and wash them for 1 min in each of the first three wells of SIM.
8. Transfer the flies to the fourth well of SIM and dissect the ovaries of all the flies (Figure 1A).
  a. Turn the female on her back and, using forceps, pull at the ovipositor to remove the posterior of the abdomen.
  b. If the ovaries do not remain attached to the posterior of the abdomen as you pull away, lightly pinch the abdomen and they will pop out.

**CRITICAL:** If combining live imaging with RNAi or other genetic perturbation that disrupts GSC/ovary function, then the ovaries may appear underdeveloped, shrunken and fibrous. This is most likely due to the absence of later stage egg chambers resulting in smaller ovarioles that are essentially just the individual germaria encased in the fibrous muscle sheath. These can still be dissected in the same way but with greater care as the germaria can be easily lost.

9. Transfer the ovaries to a final well of SIM for microdissection of individual ovarioles.
10. Using an eyelash tool, comb through the ovaries to break through the overlying muscle sheath and tease apart the individual ovarioles (Figure 1A).

***Note:*** We find that an eyelash glued to a toothpick with nail varnish provides an ideal tool for microdissection of the ovarioles; strong enough to break through the muscle layer but not too firm as to damage the tissue.

11. Sever the muscle sheath connected at the anterior tip of the terminal filament or most posterior egg chamber if necessary, using the eyelash tool or dissection scissors. Make sure to remove as much muscle sheath as possible.

**CRITICAL:** Many ovarioles will remain tightly ensheathed by the overlying muscle layer. In this case the ovarioles are held tightly together (Figure 1B). Once removed, the ovarioles appear elongated and stretched out. Only select ovarioles for imaging that are easily removed from the muscle sheath to avoid inducing mechanical stress.

12. Transfer 5–10 ovarioles in 10 μL of SIM to the dissection dish well containing 200 μL fSIM.
13. Pipette the ovarioles in 5–10 μL fSIM onto a 35 mm FluoroDish glass bottom dish and spread the droplet thin with the eyelash tool. This helps stop the ovarioles floating away from the glass bottom.
14. Try to position the ovarioles using the eyelash tool so they are close together.

***Note:*** This is not essential but helps when searching for germaria on the microscope. Repositioning the ovarioles may be difficult given the delicacy of the tissue and media surface tension.

15. Carefully add 1–2 μL thrombin (10 U/mL) dropwise (0.5 μL at a time) to form the fibrinogen-thrombin clot. Troubleshooting 1.

***Note:*** It is important to minimize contact between the pipette tip and media when adding the thrombin, as the clot begins forming rapidly upon mixing.

16. Cover the FluoroDish and leave for 10 min to allow clot formation at 20°C.

**CRITICAL:** Make sure you allow the clot long enough to solidify. There is a subtle increase in opaqueness upon clot formation.

17. Add 100 μL of iSIM prewarmed to 25°C and apply a piece of damp tissue paper to the edge of the dish to prevent drying out. Troubleshooting 2.

### Live confocal microscopy

**Timing: 2–3 h**

In this section, the microscopy setup and settings are outlined to visualize cytoskeletal projections in the germline.

18. For imaging experiments, we use a Leica TCS SP8 AOBS inverted confocal microscope and imaging is carried out at 25°C.
  a. Use a HC PL APO CS2 motCORR 63×/1.2 water objective and 1.5× optical zoom
  b. Set the pinhole to 1 airy unit and scan speed to 400 Hz unidirectional and acquire an image of 512 pixels per line and 256–512 lines at 8 bit. Acquiring an image at 512 × 512 pixels with these settings resulted in a pixel size of 241 nm × 241 nm and an image size of 123 μm × 123 μm.
  c. Use the 488 and 561 nm laser excitation lines. Collect images using the hybrid detectors with the white laser set to 70% with 488 nm (10%) and 574 nm (5%) with 2× line averaging. Troubleshooting 3. Troubleshooting 4.
    ***Note:*** The settings described here are used to image LifeAct.GFP (488 nm) and H2B.mRFP (574 nm).
  d. Set the z stack to be acquired continuously at 0.75 μm intervals of 10–20 z stacks making sure to contain all of the germarium or anterior germarium. This will allow a final temporal resolution of 30–60 s to be achieved.

19. Locate germaria on the glass coverslip using brightfield illumination and pick a germarium that is lying relatively flat to the coverslip. Troubleshooting 3. Troubleshooting 4.
20. Use fluorescence illumination to identify the GSCs and the niche cells (in this example, by their anterior localization and the absence of transgene expression or, if using a fluorescent histone marker, by their compact nuclei relative to the germ cells) and ensure the niche cells are oriented orthogonal to the GSCs. Troubleshooting 5.
21. Set up a time lapse for up to 3 h.

***Optional:*** Depending on temporal resolution restrictions, it is also possible to image multiple fields of view to image more than one germarium at a time.

### Image processing

**Timing: 30 min**

**Figure 2:**
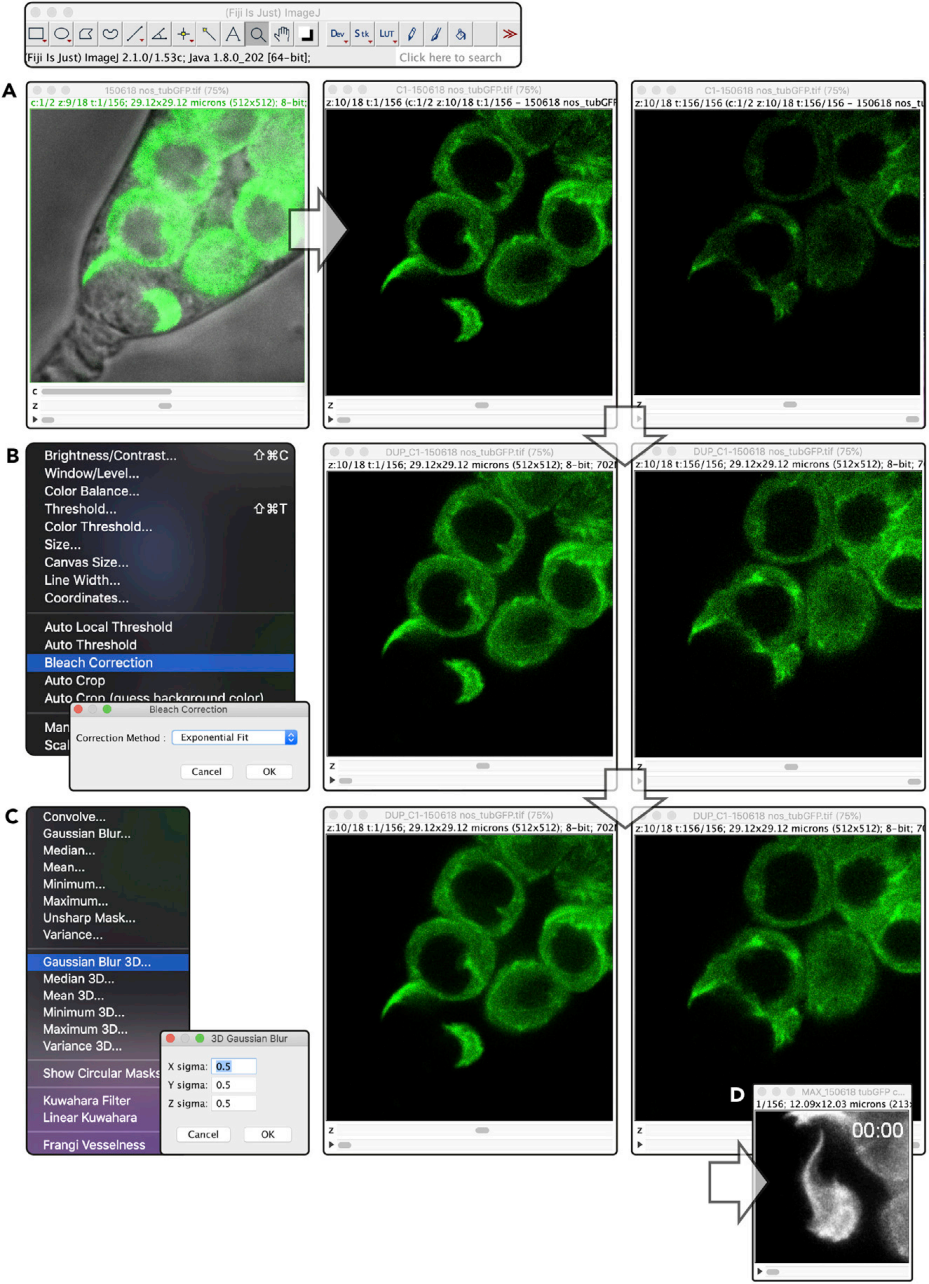
Image processing (A) Open images in Fiji. Displayed in center and right are single *z* slices at the beginning and end of the movie. (B) Adjust the brightness and apply a bleach correction. (C) Apply a 3D Gaussian blur. (D) Create a maximum projection *z* stack and crop as appropriate. See Methods video S1 in Wilcockson and Ashe (2019).
23. Adjust the brightness and apply an exponential fit bleach correction (Figure 2B).
24. Apply a 0.5x × 0.5y × 0.5z pixel-wide 3D Gaussian blur (Figure 2C).
25. Create a maximum projection *z* stack to encompass the region/structure of interest.
  a. In Figure 2D a *z* stack of the first 10 *z* slices of an 18 *z* slice movie is generated so as not obscure the view of the eGFP-Tubulin labeled GSC projection.
26. Crop the image as appropriate by drawing a rectangle around the region of interest and using the crop function (Ctrl+Shift+x).
27. Measuring projection length:
  a. Use the line tool to draw over the length of the projection and use Analyze > Measure to determine the length of the projection.
28. Save Movies as .avi files with the desired number of frames per second (for example, movies used here are either progressed at 5 or 15 frames per second).

***Note:*** Further information regarding the analysis of the germ cell projections, GSC signaling levels and GSC-niche adhesion can be found in Wilcockson and Ashe (2019).

22. Open movies in Fiji using Bio-Formats Importer with the settings below (Figure 2A):
  a. View stack with: Hyperstack.
  b. Color mode: Composite.

## Expected outcomes

**Figure 3:**
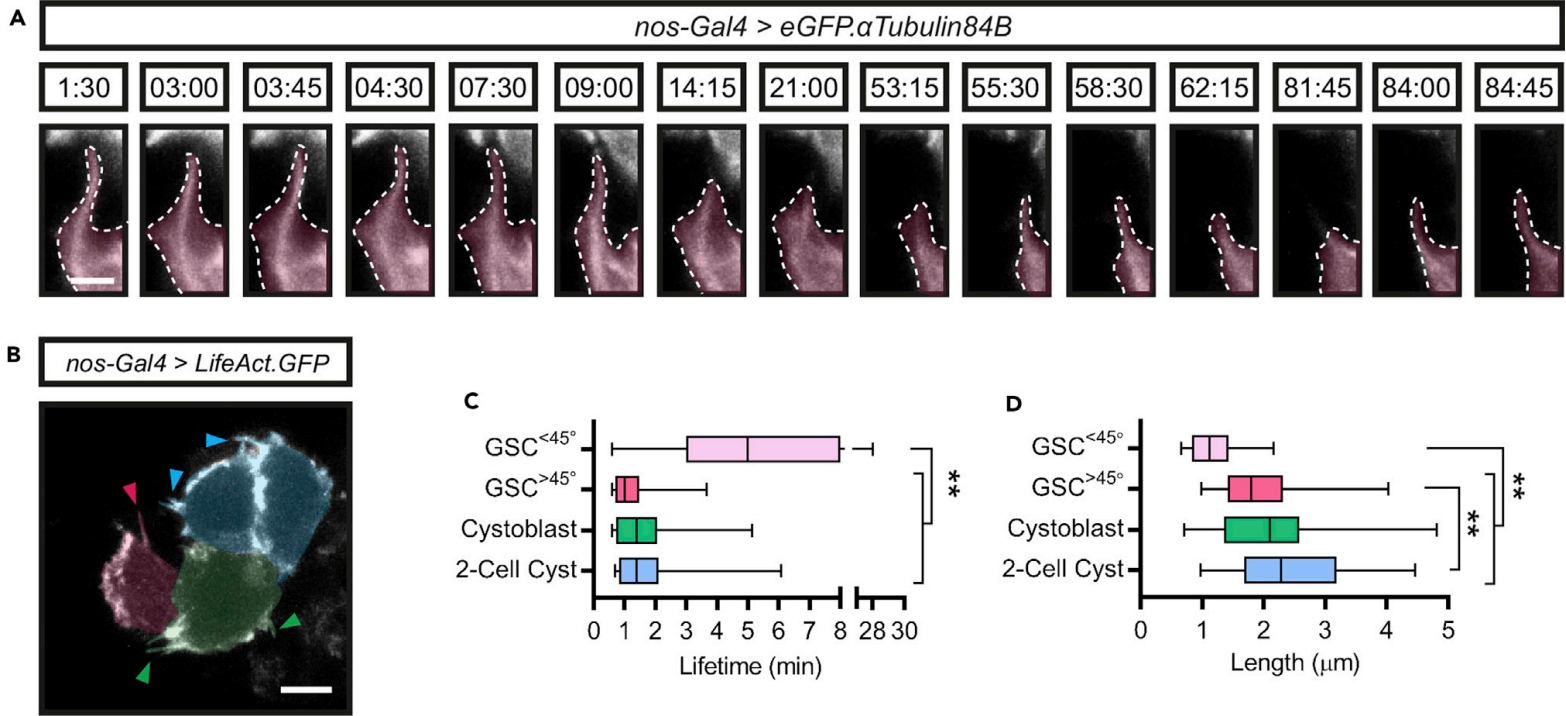
Live imaging of cytoskeletal projection dynamics in the germline (A and B) Stills from Methods videos S1 and S2 in Wilcockson and Ashe (2019) showing a microtubule-rich projection labeled with eGFP.αTub and actin-rich projections labeled with LifeAct.GFP in GSCs (false colored magenta), CBs (green) and 2- or 4-cell cysts (blue). Time is in min:s. Arrowheads indicate the tips of filopodia. (C and D) Box and whisker plots comparing the lifetime (C) and length (D) of actin projections from the following classes: GSC (magenta), CBs (green), and 2-cell cysts (blue). Dashed lines outline GSC projections. Scale bar, 2 μm (A) or 5 μm (B). See Figure 2 in Wilcockson and Ashe (2019) for further details.

Mounting ovaries in a fibrinogen-thrombin clot greatly reduces tissue movement to enable higher temporal resolution when imaging dynamic processes. In Wilcockson and Ashe (2019), we utilized this method to specifically investigate the interaction between GSCs and their associated niche cells, the cap cells (CpCs). We found that GSCs extend microtubule-rich cytocensor projections in between the CpCs (Figure 3A) while the GSCs and their differentiating daughters extend many dynamic actin-rich cytoskeletal projections (Figure 3B). The temporal resolution achieved enabled a comparison of germ cell actin filopodia dynamics (Figures 3C and 3D).

**Figure 4:**
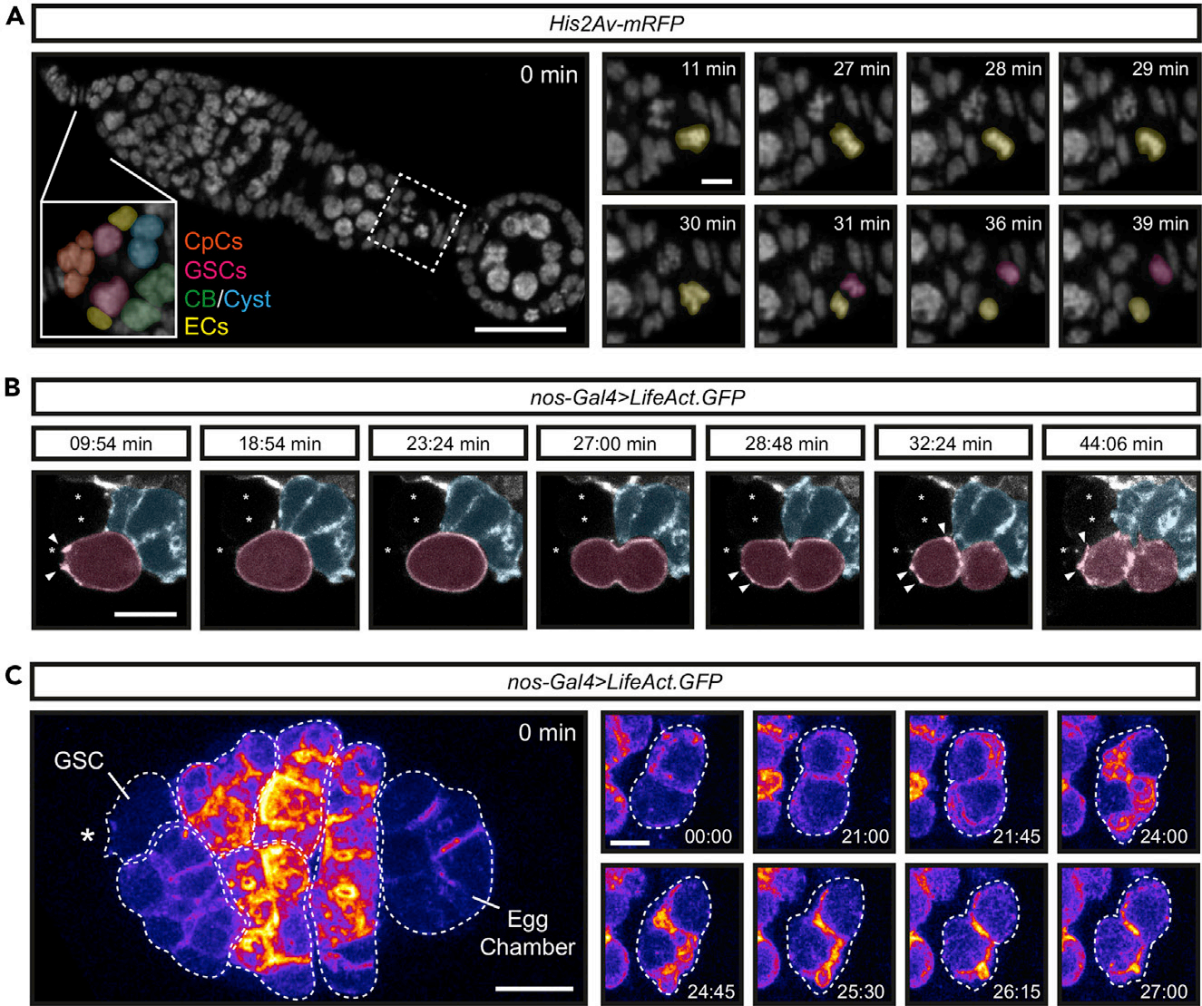
Live imaging of cell division in germ cells and the ovarian soma (A) Stills from Methods videos S1 and S2 showing the His2Av-mRFP labeled nuclei of the ovarian germline and soma. (Left inset) False coloring of the anterior germarium to highlight the different cell types of the germline stem cell niche (CpCs, cap cells; GSCs, germline stem cells; CB, cystoblast; ECs, escort cells). (Right) Stills from Methods video S2 of the area highlighted by a dashed box showing a dividing follicle cell in a budding egg chamber. (B) Stills from Figure S3 in Wilcockson and Ashe (2019) showing a dividing GSC expressing LifeAct.GFP. Arrowheads point to niche directed actin filopodia. Asterisks indicate niche cells. (C) Stills from Methods videos S3 and S4 showing germline-specific expression of LifeAct.GFP in differentiating germ cysts (different cell types are outlined with dashed lines). Time is in min:s. Germ cysts undergo waves of cortical F-actin accumulation associated with membrane blebbing. (Right) A partial stack showing two cells of a 16-cell cyst. False colored as a heatmap to highlight regions of high F-actin. Scale bar, 25 μm (A), 10 μm (C) or 5 μm (B and insets).

This technique could also be extended to monitoring other dynamic cellular processes, such as cell cycle progression in the proliferative somatic cells of the ovary (Figure 4A) and the slowly dividing germline (Figure 4B). We have also noted distinct actin dynamics in the later stage germline cysts, where waves of cortical F-actin are associated with cortical blebbing (Figure 4C). A recent study, using ovarioles immobilized in hydrogel, showed that these contractile actomyosin waves play a role in germline cyst encapsulation which is necessary for egg chamber development (Chanet and Huynh, 2020). Therefore, the method of ovariole immobilization in a fibrinogen-thrombin clot can be adapted to investigate the cell biology regulating the later germline and soma of the ovary.

Methods video S1. Ubiquitously expressed His2Av-mRFP enables live imaging of nuclear dynamics in the ovarian germline and soma, related to step 28 and Figure 4 Movie represents a maximum projection over 3 μm. The movie progresses at 5 frames per second. with frame intervals of 1 min.

Methods video S2. Follicle cell division, related to step 28 Movie is a zoomed-in view of Movie 1 focusing on the posterior region of a budding egg chamber, related to Figure 4. Movie represents a maximum projection over 3 μm. The movie progresses at 5 frames per second. with frame intervals of 1 min.

Methods video S3. Differentiating germline cysts undergo waves of F-actin accumulation and cortical blebbing, related to step 28 Germline expression of UASp-LifeAct.GFP, related to Figure 4. Movie represents a maximum projection over 3 μm. The movie progresses at 15 frames per second with frame intervals of 45 s.

Methods video S4. Two cells of a germline cyst undergo waves of F-actin accumulation and cortical blebbing, related to step 28 Movie is a zoomed-in view of Movie 3, related to Figure 4. Movie represents a maximum projection over 1 μm. The movie progresses at 15 frames per second with frame intervals of 45 s.

We believe that the fibrinogen-thrombin clot method provides several advantages over other live imaging techniques. In particular, the technical simplicity of tissue mounting and immobilization, and the low cost of reagents make this method very accessible. This method also achieves an enhanced degree of immobilization in comparison with low-melt agarose which, in our hands, is insufficient to completely immobilize the very minute tissues of the ovary. The permeability of the clot also lends this technique well to pharmacological manipulation (Hannaford et al., 2019).

## Limitations

While the method outlined here has been used for short-term imaging (2–3 h) of the germline, it has not been tested for long-term (≥6 h) *ex vivo* culture of the ovary. It has been noted in one study of *ex vivo* live imaging of the germarium encased in Matrigel, that removal of the muscle sheath is associated with reduced rates of egg chamber budding from the posterior of the germarium (Reilein et al., 2018). While we have not found this to be a problem, this could be remedied by mounting the ovarioles in their muscle sheath. The movement will be reduced by encasing the ovarioles in the fibrinogen-thrombin clot but it cannot be blocked completely and the contractions of the muscle will disrupt imaging.

## Troubleshooting

### Problem 1

The fibrinogen-thrombin clot sticks to the pipette tip and pulls away from the glass coverslip.

### Potential solution

The fibrinogen-thrombin clot starts to form immediately upon mixing the two solutions together. Therefore, to avoid the clot attaching to the pipette tip, expel the thrombin droplet from the pipette tip and allow it to contact the fSIM. As soon as the droplet of thrombin mixes with the fSIM, rapidly remove the pipette tip to stop it contacting the fSIM. Do not mix the solutions by pipetting.

### Problem 2

The fibrinogen-thrombin clot lifts away from the coverslip when adding media or does not form at all.

### Potential solution

This could be due to issues with the mounting. One possibility is that the fibrinogen concentration is too low or the fibrinogen has precipitated out of solution. This results in weaker clot formation that is easily disturbed. If this occurs remake the fSIM and ensure that all the fibrinogen has dissolved. Fibrinogen is not soluble in water so should either be dissolved in 1% saline solution or directly in SIM at 37°C with gentle agitation. Do not vortex.

Another possibility is that the clot has not been given enough time to form. Although the clot forms immediately upon mixing of fibrinogen and thrombin, in our hands we find that it has to be left for 10–15 min so that it remains firmly attached to the coverslip. It is also helpful to add the thrombin dropwise at 2–3 different points around the germaria if they are widely spread. The clot will only form so far and by adding another drop of thrombin, the area of the clot will be extended.

Finally, if the clot does not form at all then there is likely an issue with one or more of the reagents. For example, the fibrinogen has precipitated out of solution. If this problem persists then remake all of the solutions.

### Problem 3

Photobleaching occurs during live imaging.

### Potential solution

Microscope settings will need to be optimized for each fluorescent reporter. To address this, decrease the laser power and/or reduce the sampling frequency.

### Problem 4

During live imaging, the germaria move out of focus.

### Potential solution

This could be an issue with the dissection of the ovarioles. If any muscle remains attached to any of tissue on the coverslip it can cause movement throughout the clot during contractions. It is therefore essential to try and remove as much of the muscle overlying the ovaries as is possible.

Another possibility is that the clot has become detached from the coverslip, leading to the gradual drifting of the germaria away from the field of view. To address this, see Troubleshooting 2.

### Problem 5

The niche cells cannot be located.

### Potential solution

This is made easier by using markers for the somatic cells of the germarium, although there are no CpC-specific markers currently available to the best of our knowledge. There are, however, more general germarial somatic cell markers (e.g., *bab1-Gal4* labels all anterior somatic cells of the germarium, including CpCs, and *c587-Gal4* labels escort cells and follicle cells.) However, the use of these transgenic lines alongside germline-driver lines is not possible. Alternatively, the niche CpCs can be easily identified with a H2B-labeled transgene (Figure 4A) by their compact nuclei, pseudostratified arrangement, and their juxtaposition to the GSCs, which have much larger diffuse nuclei.

A general issue that arises is that the niche is not oriented exactly orthogonal to the coverslip/the germarium lengthways. This is because it can protrude away from the terminal filament cells resulting in the niche and the GSCs being oriented at an angle, with respect to the coverslip/the length of the germarium. Depending on how the tissue has oriented during the mounting stage, this may make it difficult to image both the GSCs and the niche cells, in which case, it is better to search for another ovariole on the dish to image.

## Resource availability

### Lead contact

Further information and requests for resources and reagents should be directed to and will be fulfilled by the lead contact, Hilary L. Ashe (Hilary.Ashe@manchester.ac.uk).

### Materials availability

No new materials were generated for this study.

### Data and code availability

This study did not generate any unique datasets or code.
